# Influence of Laser Process Parameters, Liquid Medium, and External Field on the Synthesis of Colloidal Metal Nanoparticles Using Pulsed Laser Ablation in Liquid: A Review

**DOI:** 10.3390/nano12132144

**Published:** 2022-06-22

**Authors:** Abdul Subhan, Abdel-Hamid Ismail Mourad, Yarub Al-Douri

**Affiliations:** 1 Mechanical and Aerospace Engineering Department, United Arab Emirates University, Al-Ain P.O. Box 15551, United Arab Emirates; 700036551@uaeu.ac.ae; 2 National Water and Energy Center, United Arab Emirates University, Al-Ain P.O. Box 15551, United Arab Emirates; 3 Mechanical Design Department, Faculty of Engineering, El Mataria, Helwan University, Cairo 11795, Egypt; 4 Engineering Department, American University of Iraq-Sulaimani, Sulaimani P.O. Box 46001, Kurdistan Region, Iraq; yaldouri@yahoo.com; 5 Department of Mechatronics Engineering, Faculty of Engineering and Natural Sciences, Bahcesehir University, Besiktas, Istanbul 34349, Turkey

**Keywords:** pulsed laser ablation, colloidal, nanoparticles, physiochemical interactions, laser process parameters

## Abstract

Pulsed laser ablation in liquid, used for nanoparticle synthesis from solid bulk metal targets (a top-down approach), has been a hot topic of research in the past few decades. It is a highly efficient and ‘green’ fabrication method for producing pure, stable, non-toxic (ligand-free), colloidal nanoparticles, which is often challenging using traditional chemical methods. Due to the short time scale interaction between the laser pulses and the target, it is difficult to achieve complete control on the physical characteristics of metallic nanoparticles. Laser process parameters, liquid environment, and external fields vastly effect the shape and structure of nanoparticles for targeted applications. Past reviews on pulsed laser ablation have focused extensively on synthesising different materials using this technique but little attention has been given to explaining the dependency aspect of the process parameters in fine-tuning the nanoparticle characteristics. In this study, we reviewed the state of the art literature available on this technique, which can help the scientific community develop a comprehensive understanding with special insights into the laser ablation mechanism. We further examined the importance of these process parameters in improving the ablation rate and productivity and analysed the morphology, size distribution, and structure of the obtained nanoparticles. Finally, the challenges faced in nanoparticle research and prospects are presented.

## 1. Introduction

**Figure 1:**
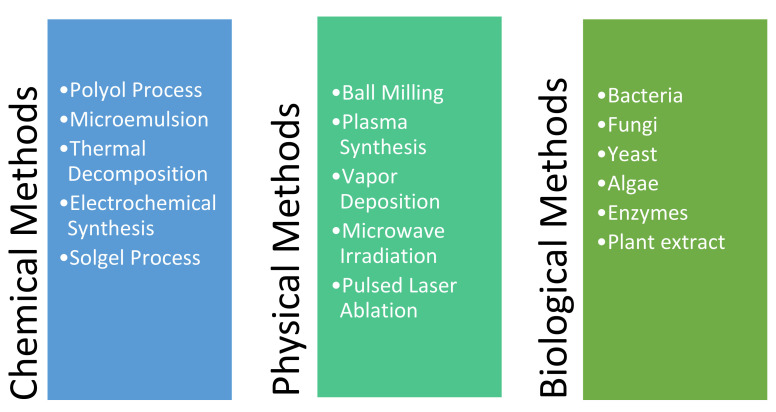
Different methods found in the literature for synthesising metal nanoparticles.

Owing to their various functions, applications, and unique physiochemical properties, nanoparticles (NPs) have attained significant technological attention in the past [1]. Because of their larger surface area to volume ratio, NPs are highly reactive and provide an excellent depiction of catalytic, optical, physicochemical and magnetic properties relative to bulk materials [2]. In the viewpoint of application, metal NPs have been extensively used in various industrial applications, such as in nanofilters for wastewater treatment in the petroleum industry [3], heterogeneous catalysts in the chemical industry [4], photodetectors in the automotive and space industries [5,6], quantum dots in the semiconductor industry [7], and disease diagnosis and drug delivery in the medical and pharmaceutical industries [8]. They are mainly classified based on top-down (bulk material to powder or particle form) or bottom-up (atoms to nanoclusters) approaches. However, another relevant way of representing their classifications is the precise methods [9,10,11,12,13,14,15,16,17,18,19,20,21,22,23,24] (i.e., physical, chemical, and biological as detailed in Figure 1) that have been used for synthesising metal NPs.

The chemical method is a bottom-up technique that involves the use of the wet-chemistry approach for producing NPs from chemical reactions, which has been a popular synthesis approach for many years [25]. However, the use of precursors, surfactants, capping agents, and solvents for particle stabilisation is challenging and along with it are risks of residual contamination, difficulty in particle functionalisation, and further harmful effects on the environment, which remain unrealised.

Biological synthesis is a green and environment-friendly approach that uses extracts from plants (e.g., algae, fungi, and yeast), microbes, and natural sources [26]. It has been found effective in producing biocatalysts in the agriculture sector and is currently being researched for other fields of application. However, significant improvements are still needed to develop this technology for the commercial scale of NP production.

Physical methods involve producing NPs in an environment-friendly manner using a top-down approach and avoiding solvent contamination. The use of high temperature and pressure conditions assisting stabilised and controlled particle growth makes it attractive to researchers and industries. However, the challenge in using this approach is its cost-effectiveness as the manufacturing equipment and energy requirement for NP fabrication at a commercial scale are expensive.

Among the physical methods, one such technique that stands out in terms of ease of production and effectiveness in controlling particle size distribution and nanostructure growth is pulsed laser ablation. Additionally, the advantage PLAL offers is that it is the fastest way to obtain colloidal nanoparticles. It may be performed in various media (i.e., air, vacuum, or liquid) [27]. Compared with air and gaseous media, pulsed laser ablation in liquid (PLAL) medium is a highly popular technique extensively used for NP fabrication [28].

**Figure 2:**
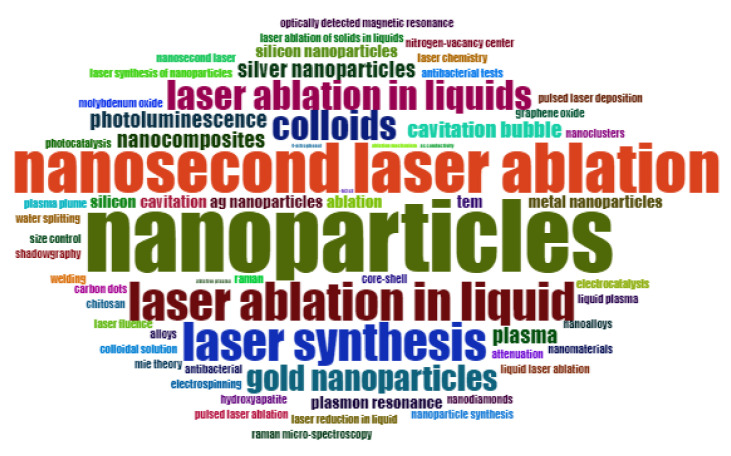
Bibliometric analysis of keywords related to pulsed laser ablation in liquid medium found in the literature using word cloud tool.

RStudio was used to perform bibliometric analysis, and then, a word cloud was generated (Figure 2), which is a descriptive visualisation of keywords found in the literature related to PLAL. The relative font size in the word cloud signifies the frequency of the keywords appearing in PLAL-related manuscripts.

The use of the pulsed laser ablation technique was first reported in 1987 by Patil et al. [29] for synthesising iron oxide NPs and, since then, the synthesis of different metallic NPs using PLAL has been used in various commercial industries, such as food, biomedical, electronics, and optical (Table 1).

PLAL has several advantages over other methods, which include reduced reaction time, the avoidance of multistep synthetic procedures, the absence of reducing agents, laboratory safety, low toxicity, and the ease of extraction because the generated NPs are confined within the liquid zone, making it an environment-friendly process [30].

Apart from its usage in the commercial industry, researchers have studied the use of PLAL in increasing the productivity of NPs. Mahdieh and Khosravi [31] reported an increase in the productivity of colloidal brass NPs synthesised using PLAL in the presence of external electric fields. Moreover, their findings encompassed surface plasmon resonance and particle size distributions and showed an increase in particle concentration due to the presence of an electric field. 

Furthermore, researchers have proven that each laser type has its unique advantages, which greatly broaden the range of choices available for synthesising different types of nanomaterials, as required. Numerous new PLAL-generated nanomaterials have been reported recently. Forsythe et al. [32] proposed the preparation of nanomaterials using pulsed laser in liquids for catalysis in water oxidation, oxygen reduction, hydrogen evolution, nitrogen reduction, carbon dioxide reduction, and organic oxidation, followed by laser-made nanomaterials for light-driven catalytic processes and heterogeneous catalysis of thermochemical processes. In their review, Fazio et al. [33] focused on laser-synthesised NPs for selected applications (i.e., sensing, biomedicine, and catalysis). Moreover, Naser et al. [34] presented the parameters affecting the size of gold NPs prepared using PLAL. Xiao et al. [35] reported the progress in external field-assisted laser ablation in liquid, an efficient strategy for nanocrystal synthesis and nanostructure assembly. Zhang et al. [36] proposed the preparation of colloidal metal NPs through laser ablation and their applications in catalysis, biology, sensing, and clean energy generation and storage. Jaleh et al. [37] reported on the recent investigations into laser-mediated synthesis of nanocomposites for environmental remediation. Al-Kattan et al. [38] analysed short-pulse lasers as a versatile tool for creating novel nano- and microstructures and compositional analysis for healthcare and wellbeing challenges, respectively.

**Table 1:** Applications of various metal-based nanoparticles used in various industrial sectors.

Reference	Metal-Based Nanoparticle	Industry	Applications
Hussain et al. (2021) [39] Hogeweg et al. (2018) [40] Mahmoud et al. (2018) [41] You et al. (2021) [42]	ZnO, SiO_2,_ CuO, Al_2_O_3_, TiO_2_ Fe_2_O_3_	Oil and gas	For enhanced oil recovery (EOR), drilling fluids, wastewater treatment
Chellaram et al. (2014) [43] Sahani et al. (2021) [44] Madkour et al. (2021) [45] Tayel et al. (2011) [46]	TiO_2_, SiO_2_ ZnO, MgO	Food industry	Food packaging and preservation, additive to improve food texture and colour, contaminant detection; flavouring powders
Díez-Pascual (2018) [47] Bhattacharyya et al. (2011) [48] Rodrigues et al. (2019) [49]	ZnO, CuO, Ag_2_O_3,_ NiO, Bi_2_O_3_, MnO_2_, Al_2_O_3_, MgO	Medical	Used in antimicrobial, antibacterial, antifungal treatment
Kong et al. (2017) [50] Gu et al. (2020) [51] Adams et al. (2014) [52]	Au, Pt, Pd. TiO, CeO	Pharmaceutical industry	Used as catalyst in drug delivery for anti-cancer, radiotherapy, gene delivery
Aliofkhazraei (2016) [53] Ali et al. (2016) [54] Tan et al. (2019) [55]	FeO_2_, Ag, CdS, GaN, Si, TiO_2_, Al	Electronic industry	Used in solar cell development, semiconductor devices, ink for 3D printing in electronics
Al tuwirqi et al. (2020) [56] Geppert et al. (2021) [57] Jiang et al. (2018) [58] Kalajahi et al. (2020) [59]	Fe_2_O_3_, ZnO,	Medical devices	Imaging & bio-analysis, metal nanoparticles doped carbon quantum dots (CQDs)
Dinca et al. (2012) [60] Rana et al. (2016) [61]	TiN SiC, Al, Fe_2_O_3_, Fe_3_O_4_, Cr	Aerospace industry	Nanoparticles as composites, surface coatings for improving the mechanical strength of aircraft structures, data storage media

Factors, such as laser ablation time and medium, affect the properties of the produced NPs [62]. Altuwirqi et al. [63] used the PLAL technique to fabricate copper (Cu) and copper oxide (Cu_x_O) NPs with a size range of 1–12 nm using spinach leaf extracts as the ablation medium to increase the oxidation and productivity of NPs. They observed a reduction in the particle size with an increase in the ablation time. Meanwhile, Du et al. [64] recognised rare-earth-activated NPs from various applications, including high-tech products, green technologies, bioimaging, and medical usage. To obtain inorganic NPs with different morphologies and sizes, PLAL is a green and versatile technique. Additionally, they investigated persistent luminescent SrAl_2_O_4_: Eu^2+^, Dy^3+^ by laser ablation in liquids and their optical features. Cui et al. [65] fabricated carbon quantum dots (CQDs) by ablating low-cost carbon cloth by ultrafast and highly efficient dual-beam pulsed laser ablation. Furthermore, the CQDs have favourable stability and excellent anti-jamming performance, which are well-suitable for cell bioimaging. Therefore, studying the PLAL mechanism is particularly compelling to increase the primary understanding of the technique, precisely because of the high interest of researchers involved in PLAL nanostructure production. Some recent reviews of PLAL [66,67,68] have discussed the importance of the purity of NPs as efficient catalysts for hydrogen production, multiplicity of targets, and different liquid environments. In their study, Reichenberger et al. [68] demonstrated the importance of laser synthesis for producing functionalised and ligand-free catalysts. However, PLAL still continues to defy researchers on having a complete comprehension. Challenges in terms of developing multi-metallic functionalised NPs and improvements in productivity and stability remain a hot topic in this field of study.

Several reviews have extensively focused on application-oriented nanostructures. Only a few review articles have focused on the essential aspects of explaining the different nanostructural formations and morphologies obtained by varying input parameters. Furthermore, the simplicity of this process seems to be misunderstood with the internal process interactions, which are far more complex in developing a complete understanding of the PLAL technique. In this context, this review was designed to present a detailed understanding of the effects of various laser parameters, the liquid environment, and external fields (i.e., magnetic, electric, and temperature) on the ablation of various solid metal targets and the resulting NP morphology, with a special focus on the challenges to engineering the desired output nanostructure.

## 2. Mechanism of the PLAL Process

PLA is a product-output-oriented process that can be used for generating NPs and in micro-machining applications [69]. Compared with the air medium, the liquid medium has shown a prominent effect on the structural formation of NPs [70].

**Figure 3:**
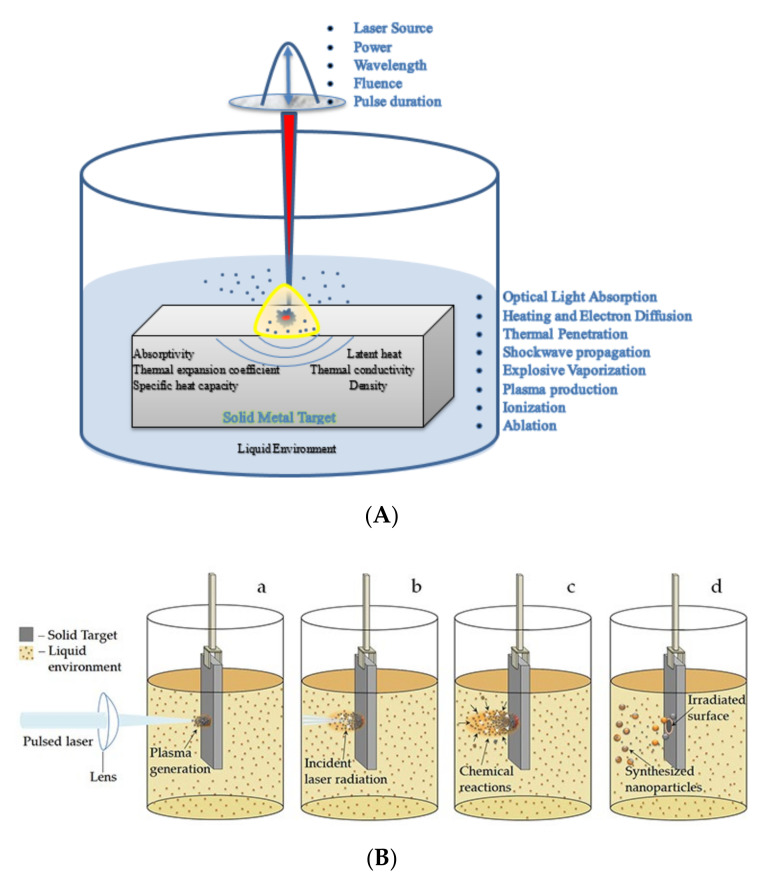
(**A**) Illustration of pulsed laser ablation in liquid technique showing the parametric influence of laser input parameters, bulk material properties, and sequential events leading to ablation. (**B**) Stages of pulsed laser ablation in liquid. Adapted with permission from Ref. [74]. Copyright 2016, intechopen.

For synthesising customised NPs, the PLAL technique was first used and reported by Nedderson et al. [71]. The PLAL process starts by directing a high-energy optical source (pulsed laser radiation) in the perpendicular direction [72,73] towards the solid bulk metal target submerged in liquid. The laser source can be directed from the top (Figure 3A) and sideways (Figure 3B).

Once the laser–matter interaction occurs, a series of thermodynamic reactions occur instantaneously, as sequenced below:

1. The absorption of radiation by the target metal surface electrons and transfer of energy to the lattice;
2. Explosive vaporisation and creation of a plasma plume;
3. The generation of shockwaves in the solution due to temperature and pressure variations;
4. The creation of a cavitation bubble expansion, shrinkage at supersonic speeds, and further diffusion, leading to the ablation of the metal target and release of NPs.

Due to the presence of a liquid medium (usually deionised water), these NPs further interact to initiate various other chemical reactions depending on the properties of the target material, type of solvent, pulse energy, and duration. Subsequently, the plasma is quenched in the liquid, leading to the formation of electrically charged NPs in metastable phases [74]. Owing to the low productivity of this process, attributed to the higher threshold limit of ablation in liquids compared to gaseous media, the adequate use of the aforementioned process parameters is a critical aspect of this field of research [75].

## 3. Physical Interactions in the PLAL Process

### 3.1. Laser–Liquid Interaction

The high-intensity laser must initially pass through the liquid medium before interacting with the target metal surface, which results in the refraction of the laser beam by ambient liquid media [75]. Hence, to achieve a desirable focus on the target, the optimum focal length should be calculated as given by [76].

(1) $$∆f=l\lfloor 1-\frac{f}{\sqrt{n^{2}f^{2}+\left(n^{2}-1\right)r^{2}}}\rfloor$$

Then, the laser intensity reduces due to dispersal by the liquid medium, interaction with the reflected beam, and secondary interactions with the already generated NPs. Light-scattering in laser attenuation systems in the different media was studied by Sulaiman et al. [77]. The concentration of the solution can be calculated by measuring the absorbance using the Beer–Lambert law.

(2) $$A=\varepsilon cl_{o}$$

### 3.2. Radiation Absorption and Energy Transfer to the Lattice

When laser-induced photonic energy strikes the metal surface, inverse bremsstrahlung (IB) [78] occurs, where radiation absorption results in the vibration of the electrons, which are ejected because of the formation of superheated plasma at temperatures of 4000–6000 K [79] and explosive solid–vapor phase transition. The plasma further expands adiabatically to create a shockwave at supersonic speeds, followed by rapid quenching and confinement due to the presence of a liquid environment. It is further shown that pulse duration (i.e., femtosecond, picosecond, and nanosecond pulses) affects the energy absorption by electrons and lattice heating time using a two-temperature thermal-optical model [80]. 

### 3.3. Cavitation Bubble Dynamics

**Figure 4:**
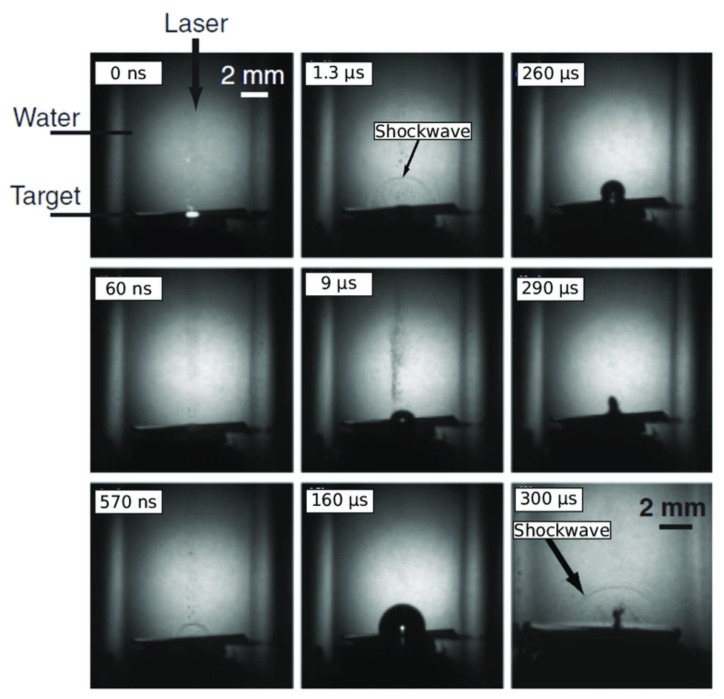
Shadowgraph images of pulsed laser ablation of Ag in liquid showing optical emissions at 0 ns, shockwave generation at 60 and 570 ns, and cavitation bubble motion (i.e., generation, shrinkage, and collapse) at 1.3–300 µs. Adapted with permission from Ref. [81]. Copyright 2007, The Japan Society of Applied Physics.

Studies [81] have focused on the dynamics of cavitation bubble formation, shrinkage and diffusion; some aspects of this thermodynamic process remain to be fully understood, such as the boundary layer interactions between the profile of the metal target and the liquid, surface tension, and pressure drop. Because these changes occur at a rapid level (in a few micro-nanoseconds), it is assumed that the bubble expands and collapses due to inertial cavitation and depends on the viscosity of the liquid. The shadowgraph technique (Figure 4) has been used to record the bubble dynamics; however, due to gas, vapour, and liquid media, as well as the refractive index being different in each medium, accurately predicting the thermodynamic behaviour of the bubble is impossible. It is further shown that the impact of the laser pulse energy contributes to bubble growth.

Huang et al. [82] examined the bubble dynamics in terms of three oscillations and found that the velocity and pressure variations inside the bubble led to their expansion and shrinkage. They reported that the expansion velocity decreases moderately with the oscillations and the shrinkage velocity increases immediately after each oscillation. However, why the oscillations in the bubble appeared thrice before releasing NPs remains to be explained. Senegačnik et al. [83] demonstrated that using the diffused illumination technique, the fluid dynamics of the bubble growth, expansion, and shrinkage can be examined more accurately. Dell Aglio et al. [72] explained the PLAL process using a time-resolved diagnostic technique and it was shown that during bubble collapse and diffusion, the NPs were released into the surrounding liquid, forming a colloidal solution. Reich et al. [84] used visible light stroboscopic imaging and X-ray radiography to investigate the bubble dynamics and found that the solid–liquid interface was a crucial factor in bubble motion and contributed to the particle isolation and withdrawal force to create a secondary collage of NPs. Moreover, changes in the liquid layer thickness have been shown to change plasma dynamics. Nguyen et al. [85] investigated this point in their study and concluded that if the liquid layer equates with the plasma size, a portion of the plasma forms in the air. However, if it is thicker, plasma confinement occurs, causing the cavitation area to be in the free boundary region [86].

### 3.4. NP Formation and Release

To predict the formation of NPs, various theories have been presented [87,88,89]. Giacomo et al. [90] examined plasma cooling using fast imaging and emission spectroscopy techniques. It was shown that plasma intensities and temperature increased rapidly and further decreased at an exponential rate. This rapid quenching phenomenon leads to an energy transfer from the plume to the surrounding liquid (of high thermal capacity), leading to an instant condensation and nucleation growth, leading to the formation of NPs.

To understand the physics behind the nucleation time and growth velocity of nano-diamond particles by PLA of graphite targets in water, Wang et al. [91] proposed a theoretical kinetic approach and validated with experimental work that the isothermal nucleation time ranges between 10^−10^ second and 1 nanosecond. Barbero et al. [92] investigated the nucleation and aggregation stages of metallic NPs in a colloidal solution during laser ablation. They explained that the nucleation mechanism is based on atoms evaporating from the sample surface during the first microsecond of irradiation and then nucleating into a plasma plume.

Taccogna et al. [93] presented a kinetic approach based on embryo growth through explicit sequential adhesion of ions by coupling between granule charge and plasma plume dynamics, resulting in the further aggregation and evaporation of atoms. However, an explanation of the mechanism of the formation of NPs remains elusive. It is assumed that vapour condensation has a dominant effect on the birth and growth of NPs, and electron-ion combination in the confined plasma induces a chemical reaction that dictates the size of NPs.

Once the ablated mass (newly formed NPs) gathers near the bubble surface, their volume increases due to aggregation, thereby exerting pressure on the bubble itself (Figure 4). Bubble collapse induces a natural repelling force, which pushes the NPs away from the target and releases them into the solution for further interaction with the surrounding liquid medium.

A study on ZnO NPs [94] showed that the nanostructure and particle concentrations heavily rely on laser input parameters. Furthermore, particle size control could be established using the method adopted by Choudhury et al. [95], where the target geometry (Cu and Au) was confined to a limited space due to which the generated shockwave reflected from the confined boundary and interacted with the plasma plume, leading to longer nucleation and hence the formation of larger NPs.

## 4. Laser Parameters Influencing the Ablation and Synthesis of Colloidal NPs

When performing a PLAL process in metals, the properties of the target material, such as light and heat absorption, thermal diffusivity (*D_T_*), attenuation coefficient (α), the heat of vaporisation (*H_v_*), and density (ρ), dictate the laser performance [18]. Once the target material properties are understood, the laser parameters can be chosen appropriately, which are necessary to achieve the desired ablation of metals. Recently [73,74,75,76,77,78,79,80,81,82,83,84,85,86,87,88,89,90,91,92,93,94,95,96], research has taken a further step, which involved the application of external magnetic, electric, and temperature fields to examine the impact of light–matter interaction and to achieve shape control on various types of nanocrystals. Experimental studies provide a perspective on the working mechanism in PLAL but a critical review is necessary to find research gaps related to this technique. To achieve the optimal conditions of laser parameters for the desired nanostructure, no accurate model exists to date. However, with the experimental investigations using the trial-and-error method, researchers have optimised them.

### 4.1. Source and Wavelength of Laser

Laser sources used in pulsed ablation of solid materials are classified into two categories; that is, a solid-state medium uses doped crystals or glass, such as Nd:YAG or Ti-Sapphire, and the gas phase (excimer or CO_2_) uses photoemissions from unstable compounds and decomposition [96]. The selection of the laser source and its wavelength is essential in generating NPs with the desired morphology. Nd:YAG is the most common laser source that is pumped using an arc lamp or a laser diode, which produces near IR wavelengths of light at *λ* = 1064 nm with the ability to double, triple, or quadruple the frequency using optics, adding to the versatility of this solid-state source [97]. 

The simplicity of this source and the avoidance of hazardous gases make it the most popular laser source in pulsed ablation research of solid metals. Ti-Sapphire requires pumping from another laser source with a highly tuneable emission wavelength (*λ* = 650–1100 nm) and could generate tens of femtoseconds of pulse duration [98]. They can be very expensive because they require a second laser source. Excimer systems can provide pulses as short as tens of nanoseconds, making them the source of choice in PLA due to the versatility of ultraviolet light, which ablates many materials [99]. CO_2_ uses gas discharge pumping and significant cooling to produce far IR wavelengths at two frequencies, which could generate pulses of hundreds of nanoseconds, which makes their usage in high-power industrial systems [100] favoured. The effects of wavelength (*λ* = 1064 nm and 193 nm) and laser source (Nd:YAG and ArF excimer) on the structure and productivity of Pd NPs were shown by Mortazavi et al. [101].

It is shown that the Nd:YAG laser has a higher plasma temperature with an excellent spherical structure and a high production rate of NPs compared to the ultraviolet wavelength. The influence of ablation efficiency and the properties of NPs using picosecond pulse ablation in Ag, Zn, and Mg in polyurethane-doped tetrahydrofuran was studied by Schwenke et al. [102]. The use of a fundamental wavelength (*λ* = 1030 nm) yields a much higher ablated mass after the same process time than the second harmonic wavelength (*λ* = 515 nm). The influence of wavelength on size control of Pd NPs was studied by Kim et al. [103]. The average size of NPs is small but has homogeneous distribution at *λ* = 355 nm and 532 nm, whereas, at *λ* = 1064 nm, the NP size is large and non-homogeneous. Baladi et al. [104] synthesised Al NPs using PLA of an Al target in ethanol and observed higher ablation efficiency and fine NP generation at higher wavelengths (1064 nm) than at a wavelength of 533 nm.

**Figure 5:**
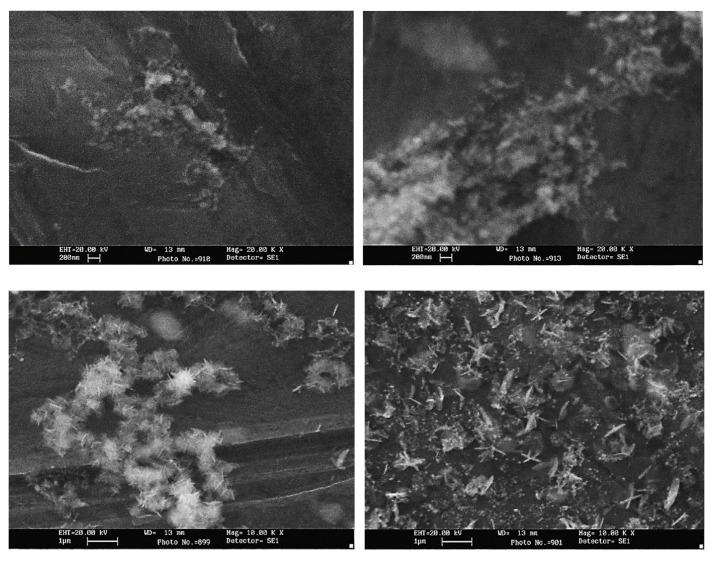
Scanning electron microscopy showing the morphology of ZnO nanoparticles at laser wavelengths of 532 nm (top row) and 1064 nm (bottom). Adapted with permission from Ref. [106]. Copyright 2013, Elsevier Ltd.

Torrisi et al. [105] compared the effects of wavelength on solid Cu using Nd:YAG (*λ* = 1064 nm) and XeCl excimer laser (*λ* = 308 nm) and showed that ultraviolet laser is more efficient in evaporating the Cu atoms, even though IR radiation has higher kinetic energy and plasma temperature. Solati et al. [106] investigated the effects of wavelength and pulse energy on the morphology of ZnO NPs in deionised water. At a pulse wavelength of 532 nm (Figure 5), they observed spherical-shaped NPs adjoined, whereas, at 1064 nm, they observed both spherical and sheet-like structures. In the case of graphene sheets, Solati et al. [107] investigated the influence of both laser wavelength and fluence on structural formation. Multilayer sheets were formed at 532 nm, and two layers were formed at 1064 nm.

It can be concluded that source and wavelength are important parameters to investigate for nanoparticle formation. Choosing the laser source with fundamental and harmonic wavelengths is critical in achieving the desired nanoparticle morphology and size distribution. Additionally, considering the cost effectiveness, some laser sources require additional cooling setup and periodic maintenance. 

### 4.2. Pulse Duration (Pulse Width)

**Figure 6:**
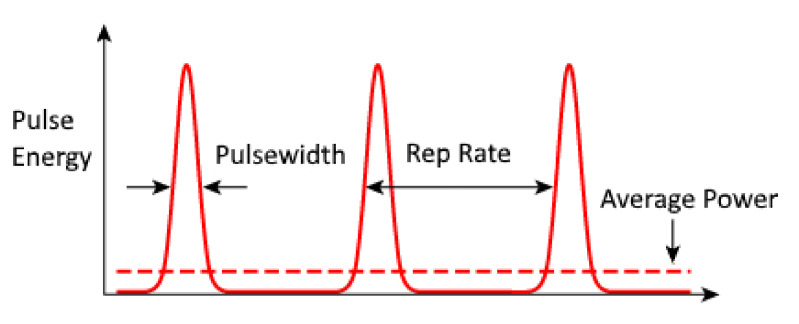
Representation of pulse width or duration.

Pulse duration (***τ***_L_), as shown in Figure 6, is the time during which the active energy is directed to the metal surface, resulting in light absorption, heat generation, and further ionisation of the metal targets. Laser ablation of solids can be mathematically modelled using a two-temperature thermal-optical model given by [35].

The optical penetration ($l_{\alpha }$) of the laser on the target material is a function of the attenuation coefficient (α) given by the following relation:

(3) $$l_{\alpha }=1/\alpha$$

The thermal penetration $\left(l_{t}\right)$ is a function of the thermal diffusivity $\left(D_{T}\right)$ of the target material given by the following relation:

(4) $$l_{t}=\sqrt{D_{T}\cdot \tau_{L}}$$

(5) $$D_{T}=\frac{k}{\rho C_{P}}$$

where *k* is the thermal conductivity, ρ is the density, and $C_{p}$ is the specific heat capacity of the target material.

The two-temperature model accounts for the energy absorption by the electrons and tracks the transfer of thermal energy to the lattice over time. The model uses the heat capacities of the electrons in the lattice.

This model uses three time scales:

Electron cooling time (*τe*)

Lattice heating time (*τp*)

Pulse duration (*τ_L_*)

(6) $$C_{e}\frac{\partial Te}{\partial t}=\frac{\partial }{\partial z}\left(k_{e}\frac{\partial Te}{\partial z}\right)-\gamma \left(Te-T\right)+\left(1-R\right)\alpha I\left(T\right)e^{-\alpha z}$$

(7) $$C\frac{\partial T}{\partial t}=\gamma \left(Te-T\right)$$

where $C_{e}$ is the volumetric heat capacity of the electron, $T_{e}$ is the electron temperature, *C* is the lattice volumetric heat capacity, $k_{e}$ is the electron thermal conductivity, γ is the electron lattice energy transfer coefficient, α is the target attenuation coefficient, *R* is the target reflectivity, and *I* is the laser intensity.

For femtosecond pulses, the energy is fully deposited before the electron cooling time passes (*τ_L_* << *τ_e_*), resulting in:

(8) $$Ce\frac{\partial T_{e}^{2}}{\partial t}=2\left(1-R\right)\alpha I\left(T\right)e^{-\alpha z}$$

Ablation per pulse is further given by:

(9) $$∆h\approx ln\frac{F}{F_{th}}\cdot \alpha^{-1}$$

For picosecond pulses, the electron cooling time is passed but not the lattice heating time (*τ_e_* << *τ_p_* << *τ_L_*), resulting in the following:

(10) $$0=\frac{\partial }{\partial z}\left(ke\frac{\partial Te}{\partial z}\right)-\gamma \left(Te-T\right)+\left(1-R\right)\alpha I\left(T\right)e^{-\alpha z}$$

(11) $$∆h\approx ln\frac{F}{F_{th}}\cdot \alpha^{-1}$$

For nanosecond pulses, the lattice heating time is exceeded (*τ_p_* << *τ_L_*), resulting in the equilibrium of the lattice and electron given by the following:

(12) $$C\frac{\partial T}{\partial t}=\frac{\partial }{\partial z}\left(k\frac{\partial T}{\partial z}\right)+\alpha Ie^{-\alpha z}$$

(13) $$∆h\approx \sqrt{D\cdot \tau_{L}}$$

Studies have shown that pulse duration can affect the structure, size, and composition of NPs with time (i.e., initial, transition, and stable phases).

The ablation of aluminium (Al) targets in distilled water was analysed at different pulse durations (i.e., 5 ns, 200 ps, and 30 fs) by Zhang et al. [108]. Moreover, the suspensions resulted in variations in colour (nanosecond pulses showed a white colour, picosecond pulses showed a light grey colour, and femtosecond pulses showed a brown colour) due to surface plasmon effects. The size of the particles significantly increases with ageing mainly due to coagulation with a spherical to doughnut-like structure observed, with a cone structure using ns pulses, a triangular structure using picosecond pulses, and a granular structure with uniform size distribution using fs pulses. Furthermore, it was shown that ultrashort pulses resulted in short and homogeneous structures.

Sakka et al. [109] observed an increase in pulse duration, which decreased the ablation rate, using ns-PLA of Cu in water. The influence of pulse duration on the mechanisms responsible for the generation of NPs at the initial stage of laser ablation was studied by Shih et al. [110]. The mechanisms are the formation of a thin transient layer between the interface of the plasma plume and liquid environment; nucleation, growth, and rapid cooling of NPs above the transient metal layer; decomposition of ablation plume below the transient layer leading to higher productivity; and broad size distribution of NPs using nanosecond PLA.

**Figure 7:**
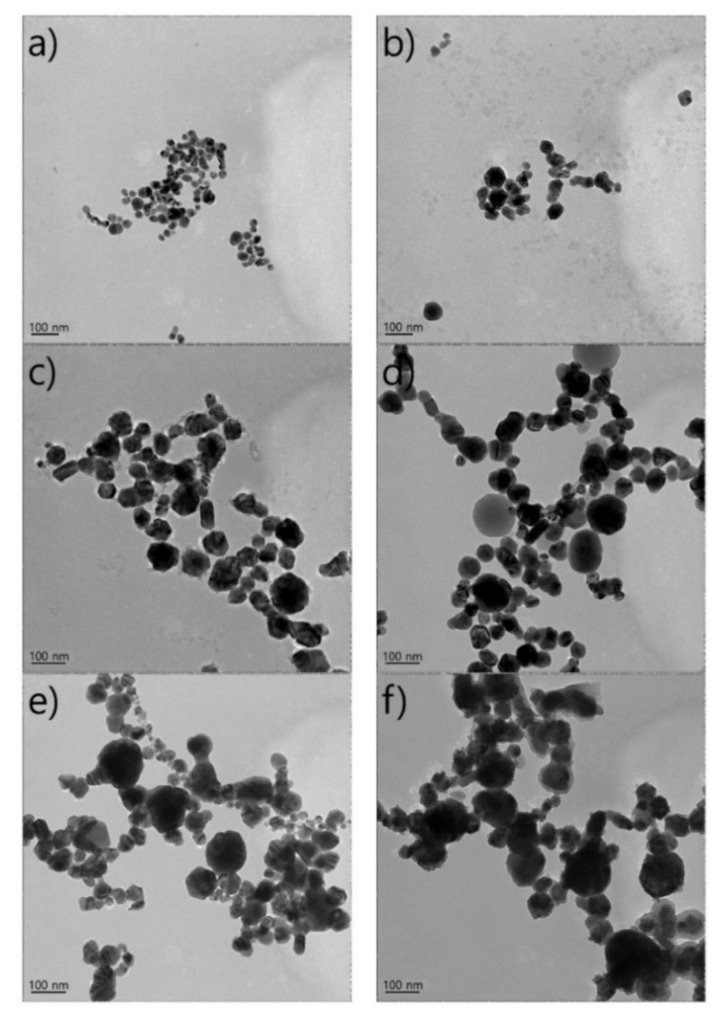
Transmission electron microscopy images showing variations in shapes of dried Ag nanoparticles using different pulse durations: (**a**) 164 fs, (**b**) 5 ps, (**c**) 4 ns, (**d**) 32 ns, (**e**) 64 ns, and (**f**) 100 ns. Adapted with permission from Ref. [112]. Copyright 2017, Printed Electronics.

Kabashin et al. [111] analysed the effects of femtosecond pulses on Au NPs in deionised water. That is, thermal free ablation leads to colloids with sizes ranging from 3 to 10 nm, and plasma-induced heating leads to a broader size distribution and larger particle size. Jeon et al. [112] studied the effects of pulse width on Ag NPs in distilled water using fs tons variation. The synthesis of the Ag NPs using fs and ps laser pulses produced a yellow solution, whereas nanosecond pulses resulted in a grey solution. Regarding size distribution, fs and ps pulses resulted in NP diameters ranging from 10 to 15 nm, whereas, using ns pulses, the particle diameter increased with sizes reaching 75–85 nm (Figure 7).

Thus, it can be concluded that pulse duration directly relates to optical and thermal penetration depths into the lattice structure and dictates the shape of the produced nanoparticles, which is highly significant. In addition, it was observed that research utilizing femtosecond and picosecond pulses are limited compared to experimental studies done using nanosecond pulses. The dynamics of an ultra-short time scale still eludes researchers to develop a complete understanding of the PLAL mechanism.

### 4.3. Laser Fluence

Laser fluence is an important parameter that influences the ablation of the metal target, which measures the amount of optical energy deposited per unit area on the material (J/m^2^). In contrast, laser intensity is the measure of the optical power per unit area (W/m^2^) related to the optical breakdown of the liquid environment. 

To ablate the material, the minimum deposition energy needed to achieve vaporisation is the threshold fluence ($F_{th})$. For short pulses, $\tau_{L}\leq 10^{-11}sec$, the volume in the heat-affected zone can be limited to optical penetration depth giving a threshold fluence independent of the pulse duration.

(14) $$F_{th}=\frac{\rho H_{v}}{\alpha }$$

For pulses $\left(\tau_{L}\geq 10^{-11}s\right)$, the thermal penetration depth exceeds the optical penetration depth; therefore, the threshold fluence grows with the pulse length.

(15) $$F_{th}=\rho H_{v}l_{t}$$

where $H_{v}$ is the heat of vaporisation.

**Figure 8:**
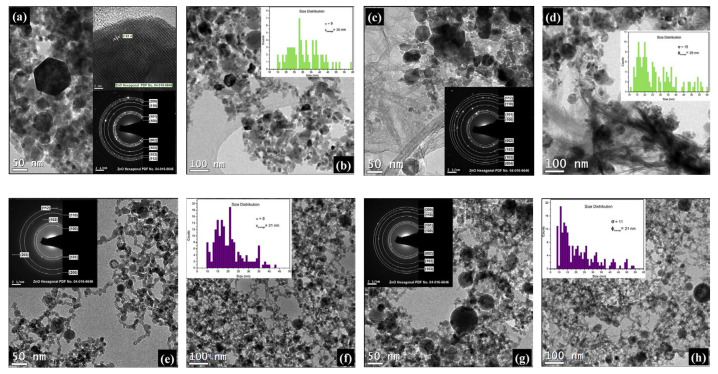
Transmission electron microscopy images of the zinc nanoparticles produced at a laser wavelength of 532 nm in distilled water (**a**–**d**) at 70 °C and (**e**–**h**) 90 °C with fluence values of (**a**,**b**,**e**,**f**) 6.0 J/cm^2^ and (**c**,**d**,**g**,**h**) 8.6 J/cm^2^. Inset images illustrate (**b**,**d**,**f**,**h**) the size distribution, (**a**,**c**,**e**,**g**) the selected area diffraction (SAED) patterns, and (**a**) the high-resolution transmission electron microscopy image. Adapted with permission from Ref. [114]. Copyright 2015, Elsevier B.V.

Research has shown that laser fluence influences NP size and distribution. Abbasi et al. [113] examined the effects of laser fluence (1–3 J/m^2^) on Al NPs generated using PLA of Al in deionised water. They observed that the size of the produced Al-oxide NPs increased with fluence at levels below the threshold value. For values above the threshold fluence, the particle size decreased. This is explained by the larger pulse energy absorption in the liquid medium. Furthermore, the ablation rate increases with its fluence. Similarly, Zn NPs were synthesised by PLAL in distilled water by Guillen et al. [114] at different fluence ranges and water temperatures. They observed different morphologies (elongated and spherical) with varying fluence rates (Figure 8).

Haram et al. [115] investigated the effects of fluence (5.73–9.87 J/cm^2^) on CuO NPs in distilled water. They observed that the width of the particle distribution and the mean size increased with fluence. Amendola and Meneghetti [116] examined Au NPs in an aqueous solution with laser fluence values varying from 12 to 442 mJ/cm^2^. They observed that with ns pulses in the range of 4–30 nm, effective control of particle diameters was possible through the mechanism of heating and rapid cooling.

Furthermore, for Au, a theoretical correlation between particle diameter and laser fluence was reported by Pyatenko et al. [117] and experimentally investigated by Tsuji et al. [118]. Laser fluence affects the optical properties and structure of CuO NPs by ablating Cu in water, which was examined by Aghdam et al. [119]. They observed the crystal growth of Cu_2_O NPs with increasing fluence. Xu et al. [120] analysed the effects of fluence on Ag colloid NPs in distilled water. They observed that the smallest mean diameter was 17.54 nm and the narrowest particle distribution was 36.86 nm at a fluence of 4.2 J/cm^2^. Colloidal NPs produced by ablating Al in ethanol are synthesised, and their effects on fluence was investigated by Mozaffari et al. [121]. They observed that at two experimental schemes (the electric field parallel and perpendicular to the laser propagation), the ablation rate of NPs increased with fluence. Al-Douri et al. [122] examined the effects of laser fluence on the size distribution of GaO NPs and observed an increased particle size with higher fluence; moreover, they reported that the particle size depended on the nature of the liquid environment. 

Hence, past research has shown that fluence is an important aspect in determining effective control on particle diameter. Additionally, the ablation rate of nanoparticles is directly related to energy density. 

### 4.4. Pulse Repetition Rate (PRR) or Pulse Frequency

**Figure 9:**
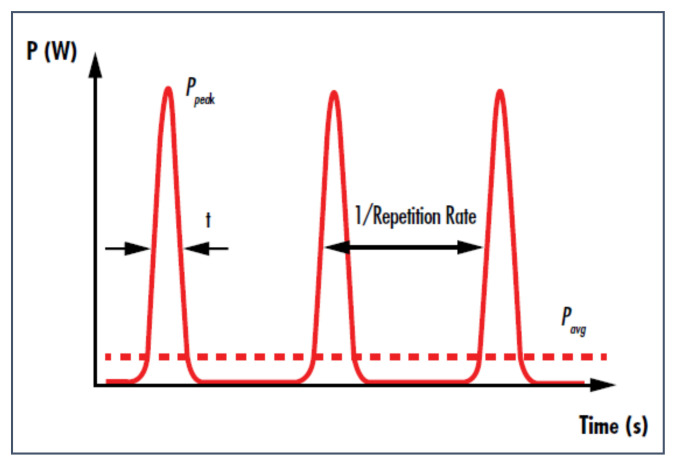
Illustration of the pulse repetition rate of a laser wave.

Pulse frequency is the number of laser pulses emitted per second (Hz) (Figure 9). The control of the pulse frequency is necessary due to the shielding effect produced by the plasma over the laser pulse, which indirectly affects the productivity of the ablated NPs. Thus, to reduce this shielding effect from the previous pulse and the successive overlap with the next pulse, variance in PRR, beam spot size and relative motion between the laser and target is required. An empirical relationship between the ablation rate and target surface condition is given by the overlapping factor [70].

(16) Of=1−VPRRS+Vτ×100

where *V* is the scanning speed, *T* is the pulse duration, and *S* refers to the laser spot size on the target.

The average laser power is given by the product of the laser pulse energy and pulse frequency.

(17) $$P_{avg}=E\cdot PRR$$

**Figure 10:**
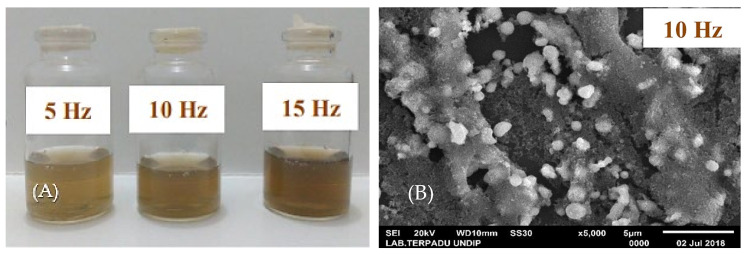
(**A**) Zn nanoparticles prepared using pulsed laser ablation of Zn in deionised water at different pulse frequencies of 5 Hz (light brown), 10 Hz (medium brown), and 15 Hz (dark brown). (**B**) Scanning electron microscopy images showing the morphology of dried Zn nanoparticles at a pulse repetition rate of 10 Hz. Adapted with permission from Ref. [124]. Copyright 2019, IOPscience.

If we increase the pulse energy (*E*), it may result in productivity losses in ablation; therefore, methods for controlling the PRR in achieving the desired ablation productivity are necessary. This can be performed by deflecting the laser using scanning and beam guidance methods for achieving full temporal pulse separation. Meanwhile, Waag et al. [123] conducted a comparative study on beam guidance methods (using galvanometric mirrors and polygon wheels) in picosecond pulsed laser synthesis of Au and Pt colloidal NPs and their alloys. They observed that the optimum laser power delivery for NP generation depended on the scanning length of the beam, which further depended on the length of the target material itself. Polygon wheel scanners are efficient in this case but at the expense of power losses. Furthermore, Sa’adah et al. [124] analysed the effects of the PRR (i.e., 5 HZ, 10 Hz and 15 Hz) on the synthesis of Zn colloidal NPs (Figure 10). It was observed that varying the PRR changed the colour of the colloidal solution, indicating that different particle size distribution and spherical shapes (single surface plasmon resonance peak) at λ=300 nm were obtained. For a PRR of 10 Hz and 15 Hz, the diameter of Zn NPs was 12.1 and 5.6, respectively.

Similarly, Alva et al. [125] investigated the effects of the laser ablation efficiency by varying the PRR (1–10 Hz) of Ag NPs in ethanol. It was shown that the productivity and ablation efficiency of Ag NPs increased with an increase in the PRR. For PLA of Ag NPs in distilled water, the effects of the PRR (20 kHz) on morphology was studied by Nikolov et al. [126] and optimal conditions were determined to achieve the highest ablation efficiency. Similarly, Ganjali et al. [127] examined how Ni NPs were produced. Thus, the significance of laser parameters in achieving control of the nanostructure and NP size is demonstrated in Table 2.

Therefore, the laser ablation rate, productivity, and ablation efficiency increase with the PRR and show an effect on the bandgap in semiconductors. The temperature and density distribution in the plume strongly depend on pulse frequency and inter-pulse separation.

## 5. Effects of a Liquid Environment on the Synthesis of NPs

The addition of reactive solutes in the liquid environment results in the chemical interaction of metal NPs with oxygen atoms. Control on the composition of NPs can be established by the nature of the solvent used because of the chemical interactivity of the plasma plume with the solvent, particularly if the restriction of the plume is limited by the viscosity/density of the solvent. Moreover, by using different solvents, the composition of the nanostructure formed can vary. Kanitz et al. [139] examined the structure and morphology of magnetic NPs using five solvents with femtosecond laser pulses and concluded that the molecular structure of the liquid medium dictates the nanostructural formation. However, the difference that femtosecond pulses make in structure formation compared to nanosecond pulses has not been explained. Ablation in water has shown higher absorption than in other liquid mediums (i.e., ethanol and acetone). Gondal et al. [140] assessed this effect on ZrO_2_ NPs and observed that the crystallite size changes due to the oxidising medium.

Furthermore, Tsuji et al. [141] showed that solvent affects the ablation efficacy and stability of NPs as well. Chemically reactive metals in water usually form oxides or hydroxides. PLA of Ni and Sn in liquid has been shown to produce core-shell type formations with a metal core and oxide layer on the surface [142]. Using femtosecond laser pulse radiation and the β-cyclodextrin aqueous solution, stable Au NPs of sizes up to 2 nm were achieved by Svetlichnyi et al. [143]. Using HCl, NaCl, and NaOH as electrolytes in an aqueous solution, stable Ag NPs were produced [144]. Amendola et al. [145] examined PLA of Fe in water that yielded a polycrystalline structure of FeO_x_ NPs, which exhibit magnetic properties and can have biomedical applications [146,147]. He et al. [148] observed the behaviour of ZnO NPs using PLA of Zn in surfactant-free aqueous solutions. An increased surface charge of NPs leads to a narrow size distribution using surfactant-free solution, and coalescence using NaCl by decreasing the surface charge was observed.

Bajaj et al. [149] investigated the effects of various liquid environments (i.e., deionised water, ethanol, and acetone) on the size and shape of Sn NPs produced using the PLAL technique and observed that the size distribution and particle size decreased with the use of surfactants. PLA of Co in different solvents (i.e., water and hexane) was examined by Tsuji et al. [150]. They observed that the formation of different compounds of Co mainly relies on the solvent. A study on the nucleation, aggregation, and growth of Au NPs in different ethanol concentrations was conducted by Tilaki et al. [151]. The effects of different solvent (i.e., polyvinylpyrrolidone [PVP]) concentrations in distilled water for Cu NPs produced using PLA were investigated by Budiati et al. [152]. They observed that the most stabilised NPs were formed at a PVP concentration of 5 mM. Similar work on PLAL to examine the effects of the liquid environment is presented in Table 3.

## 6. Effects of External Field-Assisted Pulsed Laser Ablation

Another fascinating area of research has emerged in PLAL, which involves the introduction of an external environment, such as an electric, magnetic, or temperature field, to examine the behaviour of NPs [160]. External fields have shown a dominant effect on nanostructural formation.

### 6.1. Electric Field-Assisted PLAL

**Figure 11:**
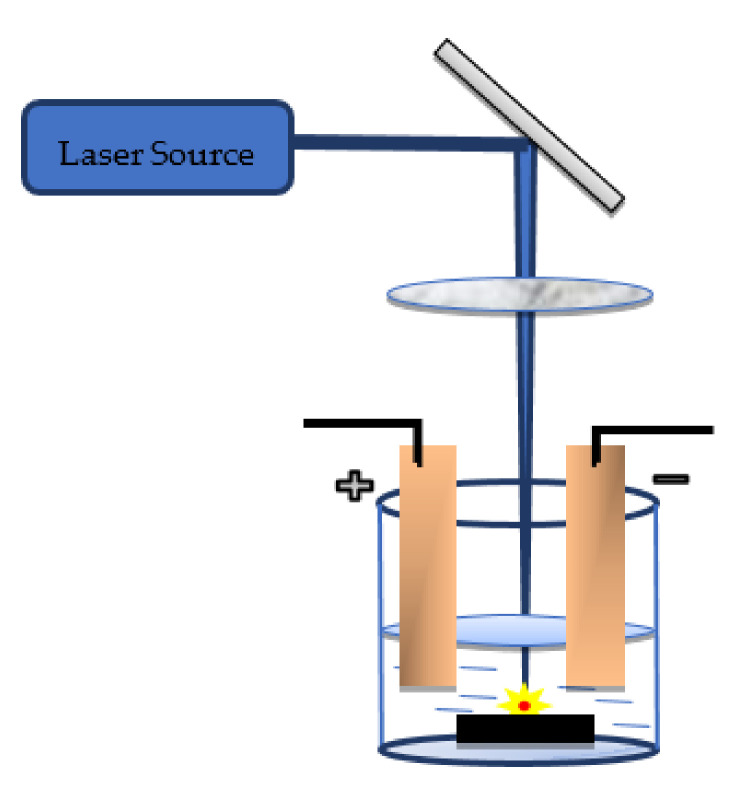
Illustration of electric field-assisted pulsed laser ablation of solid metal targets in liquid media.

The electric field significantly affects the transport of charged particles produced in the plasma to the electrodes. However, in electric field-assisted PLA, the target material does not react with the electrodes (Figure 11). Spadaro et al. [161] examined the application of electric fields to producing molybdenum oxide NPs in water. They observed a structural reorganisation in the NPs due to this influence. Meanwhile, Ismail et al. [162] analysed the effects of an electric field on the properties of Bi_2_O_3_ NPs immersed in water. They observed that the particle size increased with the application of an electric field, and complete oxidation was achieved. Lui et al. [163] synthesised GeO_2_ NPs prepared under the influence of an electric field. They proposed the growth mechanism of nanostructures by varying the electric field to form metastable structures and shapes.

Haddad et al. [164] conducted a study on the effects of a DC electric field on the synthesis of Au NPs. At low voltages (0.5–1 V/cm), the NPs are spherical and 10–18 nm in size. With an increase in voltage, various shapes (i.e., cubes, nanospindles, triangles, and rhombuses) with larger concentrations were observed. The optical and structural synthesis of Pt NPs under an applied electric field was performed by Moniri et al. [165]. They observed that the size of the NPs decreases (from 20 to 9 nm) with the formation of various shapes (i.e., rectangular, hexagonal, and rhombic), applied at larger electric voltage values. An approach for controlling the size of Sn NPs through PLAL in the electric field was proposed by Sapkota et al. [166].

**Figure 12:**
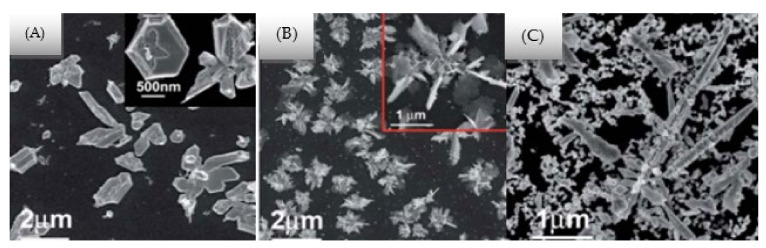
Scanning electron microscopy images of Ag films produced in Si wafer after varying the current densities at (**A**) 2, (**B**) 10, and (**C**) 200 µAcm^−2^. Adapted with permission from Ref. [168]. Copyright 2011, RSC Pub.

Mahdieh et al. [167], in their study on PLA of Al NPs under an electric field, concluded that the initial charge on the target has a significant effect on the morphology of the produced NPs. Liu et al. [168] presented a novel technique for generating varying nanostructures (i.e., nanoflower, nanoplate, and nanosalt) of Ag NPs by varying the electric current density and influence of the electrode plate (Figure 12).

### 6.2. Magnetic Field-Assisted PLAL

**Figure 13:**
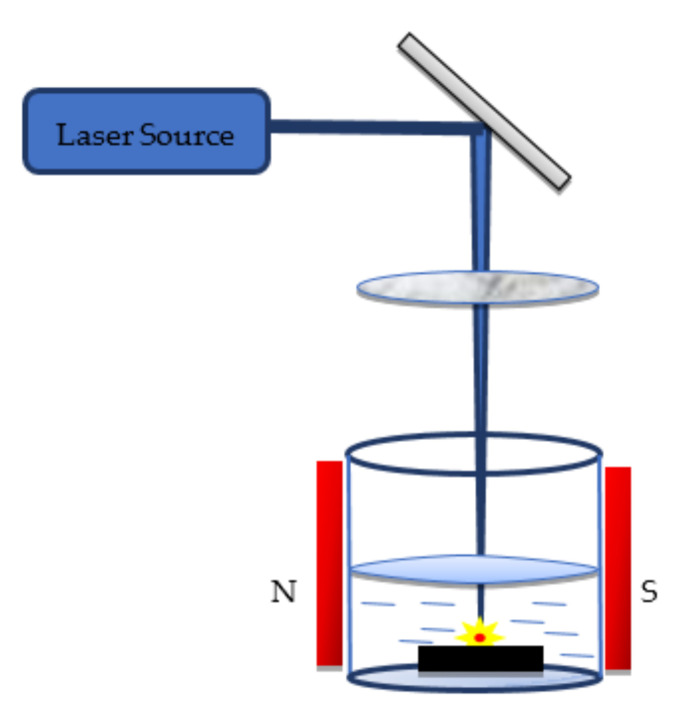
Illustration of magnetic field-assisted pulsed laser ablation of solid metal targets in liquid media.

A magnetic flux is generated by placing magnetic plates at either end of the ablation chamber (Figure 13). Studies have shown that the introduction of a magnetic field enhanced the ablation rate and optical properties of NPs. Safa et al. [169] experimentally investigated the effects of varying the transverse magnetic field on NiO NPs. The strength of the magnetic field is controlled by varying the distance between the magnets. Due to this effect, agglomeration with cyclotron motion of the particles is reduced. Meanwhile, Ghaem et al. [170] examined the effects of a DC magnetic field on Co NPs in distilled water. They observed that the particle size reduces significantly with an increase in the applied magnetic field and the formation of crystalline nanostructures. Similarly, for Ag NPs, a study was conducted by Abbas et al. [171], who observed that the average diameter of the produced particles increased from 14 nm to 25 nm by applying a magnetic field. Similarly, Kim et al. [172] observed in their study on Ag NPs that the plasma emission increases with the magnetic field. Ismael et al. [173] conducted a study on the influence of a magnetic field on synthesised iron oxide NPs immersed in dimethylformamide. Transmission electron microscopy (TEM) images confirm that the particle size reduces under the application of a magnetic field.

**Figure 14:**
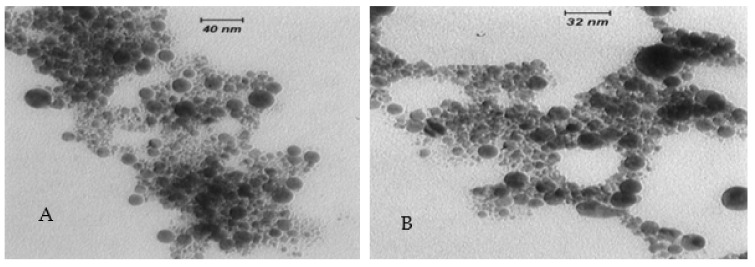
Transmission electron microscopy images of spherical-shaped Pt nanoparticles in (**A**) water and (**B**) methanol under the influence of a magnetic field. Adapted with permission from Ref. [177]. Copyright 2016, Sphinx Knowledge House.

Serkov et al. [174] examined the effects of laser-induced plasma under a high-intensity magnetic field for Au NPs. They observed that the plasma emission started earlier and increased the plume luminosity, and further accelerated the fragmentation of Au NPs. Nikov et al. [175] presented a method for fabricating micron size particles using PLA of nickel in double distilled water and ethanol under an external magnetic field. Musaev et al. [176] observed a higher fraction of spherical and shorter nanowires during the synthesis of Au NPs due to the presence of a magnetic field. This formation was because of the magnetic confinement of the plasma plume during the expansion process. The optical properties and structure of Pt NPs were examined under the application of an external magnetic field by [177]. They observed that the absorption peak is more evident, indicating an increase in the ablation efficiency due to the effect of the magnetic field and an increase in size and concentration of NPs. Figure 14 shows the agglomeration of NPs under an applied magnetic field investigated by Dahash et al. [177].

### 6.3. Temperature Field-Assisted PLAL

**Figure 15:**
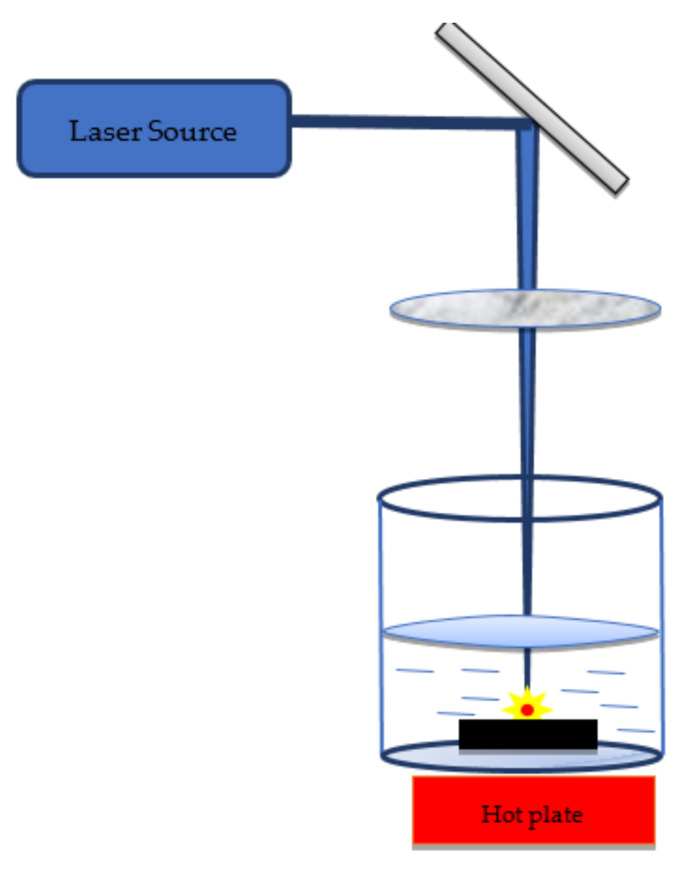
Illustration of temperature field-assisted pulsed laser ablation of solid metal targets in liquid media.

In this ablation technique, the liquid environment is heated up to a certain temperature using a hot plate (Figure 15) and its effect on NPs is examined. Solati and Dorranian [178] experimentally used this technique to investigate the characteristics of ZnO in distilled water at various temperatures (0 °C–60 °C). They observed that the size of NPs decreased while their bandgap energy increased with the rise in water temperature. Moreover, the ablation rate and crystallinity depend on the temperature. This effect is because of the dynamics of cavitation bubbles. They further estimated the lattice strain in the produced NPs and concluded that the size distribution highly depends on the temperature of the ablated environment [179]. Haram and Ahmed [180] examined the formation of Ag and Au nanochains and superclusters in distilled water at 70 °C. They observed ring-like structures and further concluded that the effect of temperature has a fusion effect on NPs, which produces nanochains and nanoclusters.

**Figure 16:**
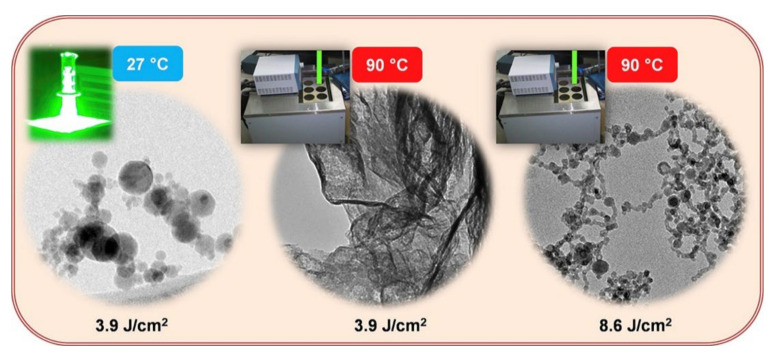
Transmission electron microscopy images of Zn nanoparticles at varying water temperatures and fluence ranges. Adapted with permission from Ref. [114]. Copyright 2015, Elsevier B.V.

Menendez et al. [181] examined the effects of water temperature on the hydrodynamic diameter and physical properties of gold NPs. They concluded that due to a reduction in the hydrodynamic particle diameter, few agglomerates are dispersed in the liquid, thereby reducing the poly-dispersity index of Au NPs. Contrary to studying the effects of increased temperature, Hong et al. [182] investigated the impact of a cooled liquid environment at room temperature. After ablation, a strong chemical reaction at the surface of the substrate occurred during rapid cooling at vaporisation temperature. Furthermore, the ablation rate decreased as the temperature was increased because of the formation of Si(OH)_x_ debris, which blocked the laser from further ablation. Guillen et al. [114] examined the structure and morphology of ablated Zn NPs by varying liquid temperatures (i.e., 50 °C, 70 °C, and 90 °C). Temperature variations led to the formation of ZnO and Zn(OH)_2_ NPs of various sizes and morphologies (Figure 16).

## 7. Bi-Metallic NPs

Because previous studies [32,33,34,35,36,37,38,39,40,41,42,43,44,45,46,47,48,49,50,51,52,53,54,55,56,57,58,59,60,61,62,63,64,65,66,67,68,69,70,71,72,73,74,75,76,77,78,79,80,81,82,83,84,85,86] focused on generating metal or metal oxide NPs from a pure bulk metal surface, a new research area in synthesising alloy NPs (bi-metallic, tri-metallic, etc.) has emerged. In this process, bulk metal alloy targets or one metal is ablated into colloidal NPs of other metals to create a hybrid combination of nanocomposites [183,184] for new applications, such as bioimaging and solar cell materials [185].

**Figure 17:**
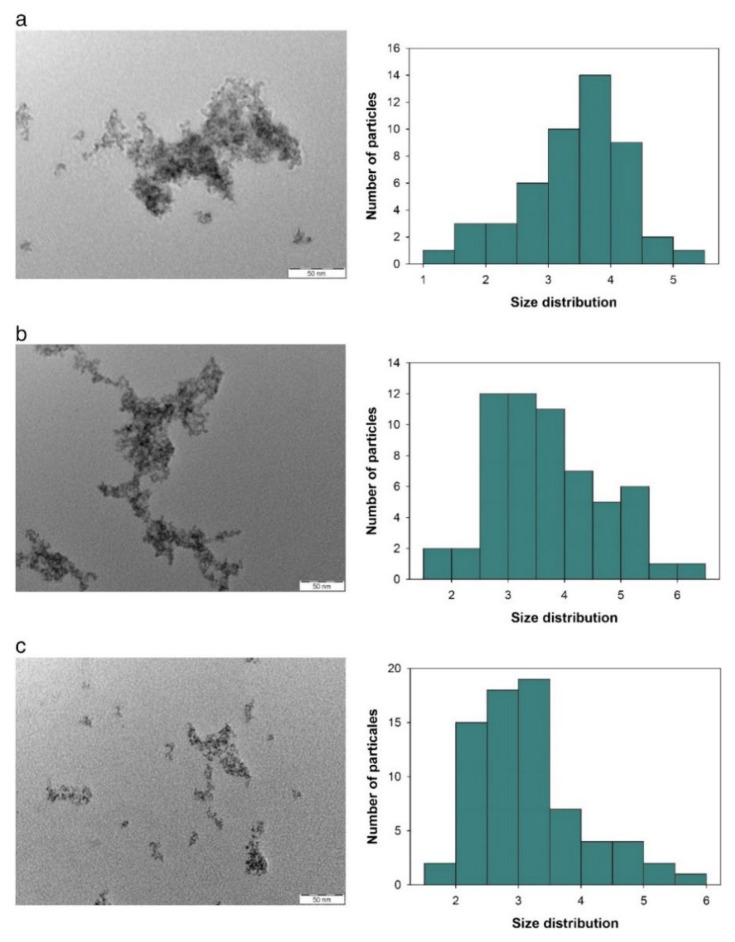
Transmission electron microscopy images corresponding to a histogram of GaN-based colloidal nanoparticles in distilled water for fluence values of (**a**) 1500 J/cm^2^, (**b**) 1100 J/cm, and (**c**) 380 J/cm^2^. Adapted with permission from Ref. [187]. Copyright 2019, Elsevier B.V.

Based on a previous study, alloy NPs have improved thermophysical properties compared to single-metal NPs [186]. To the best of our knowledge, studies in this area are limited. Al-Douri et al. [187] conducted a study by ablating GaN plates immersed in distilled water with nanosecond pulses and synthesised GaN colloidal NPs with variance in the fluence range from 380 to 1500 J/cm^2^. They observed that the mean size and productivity of the NPs increase with laser fluence because a rise in the laser absorptivity has a considerable effect on the particle size. A further increase in the fluence resulted in the interaction of the laser with colloidal NPs, leading to fragmentation and agglomeration. Figure 17 illustrates the effect of fluence variations on productivity and the mean size of the colloidal NPs.

Furthermore, Wagener et al. [188] synthesised FeAu alloy NPs by PLA of alloy metal targets in different types of solvents. They observed that in the presence of acetone, the formation of Au shells overlaps a non-oxidised iron core, and in the water medium, an Au core with a Fe_3_O_4_ shell is generated, highlighting the crucial role of solvents in the formation of the nanostructure. Neumeister et al. [189] analysed nine combinations (molar fraction) of Au-Ag alloy compositions using PLA of bulk alloy targets in liquid, leading to the formation of homogenous alloy NPs. The mixing of elements is caused by ablation in a liquid environment and re-solidification in a monophasic solid solution state.

Semiconductor quantum dots are extensively useful in fabricating photovoltaic devices. Hence, the synthesis of semiconducting nanocrystals using the PLA technique is a good approach. Sharifi et al. [190] synthesised GaAs nanocrystals using PLA of a GaAs wafer in liquid. Using this method, pure nanocrystals are obtained with a bandgap larger than the bulk composite itself. Similarly, Al alloy NPs useful in aerospace applications were synthesised by Roston et al. [191]. Similarly, nanoparticles synthesized for other applications have been reported [192,193,194,195,196,197].

## 8. Conclusions

PLAL is a fast-emerging technique for nanoparticle synthesis reported over the last years. Obtaining pure, stable, ligand-free NPs and eliminating harmful precursors makes PLAL standalone. Thus, it is not only a green technology but also economically viable over traditional chemical routes of synthesis. It is concluded that laser parameters, liquid medium, and external fields significantly impact the ablation process, effecting the morphology and size distribution of colloidal NPs. Previous works on PLAL suggest that femtosecond or picosecond pulses were better for nanoparticle generation-alienating thermal interactions compared to nanosecond pulses. Lastly, the PLAL process is agile, i.e., produces functionalized nanoparticles, which can be tailored to suit a particular application.

## 9. Prospects

Despite development in scientific research related to PLAL, several issues need to be addressed in this technique:

(1) Laser parameters can be further optimised by considering the effect of material properties that play a major role in the nanostructure formation.
(2) The physiochemical interactions occurring in the PLAL process can be further studied by analysing the thermodynamic process and the chemical reactions between the liquid environment and the ablated NPs.
(3) Further investigations in the effect of ablation angle could be performed because the available literature has focused only on the perpendicular laser beam striking the material target.
(4) Mitigating the toxic effects of colloidal NPs while retaining their most desirable optical properties remains a challenge. Hence, achieving excellent biocompatibility is a prerequisite for the widespread use of colloidal NPs.
(5) Instead of studying PLAL as a standalone technique, further integration with other physical or chemical methods can be performed to help stabilise the colloidal NPs for targeted applications. To the best of our knowledge, studies in this field are limited.
(6) From this review, we have observed that the liquid environment, material properties, laser parameters, and external field have a combined effect on the size of the obtained NPs and their distribution, morphology, and structure. However, deciding which parameter plays a decisive role in the resultant formation of the desired NPs remains a challenge. Furthermore, we found studies on PLAL [34,67,72,92,98,99,100,101,102,103,104,105,106,107,108,109,110,111] that focused on single-parameter evaluation; however, the area of multiparametric analysis of laser parameters on nanoparticle formation can be explored.

## Figures and Tables

**Table 2:** State of the art studies on different metal nanoparticles showing the influence of laser parameters on the morphological, structural, and property changes in nanoparticles.

Reference	Studied Metal/Metal Oxide Nanoparticle	Laser Parameters	Characteristic Investigated	Remarks
Alwa et al. [125]	Ag NPs	Laser wavelength (355 nm, 532 nm) Laser fluence (38.2, 76.4, 144.6 J/cm^2^)	Stability and size distribution	Spherical nanostructure NP size increased with wavelength broader size distribution with increased fluence
Ganjali et al. [127]	Ni NPs	Laser fluence per pulse (50, 100, 150 mJ)	Structural, optical, antibacterial property	Energy bandgap changed with fluence. Enhanced antibacterial activity by reducing particle size
El Faham et al. [128]	Mg NPs	Laser wavelength (1064 nm) Pulse duration: 7 ns PRR: 10 Hz Ablation time: 10–30 min	Spectral line intensities, plasma parameters	An increase in ablation time leads to a blue shift in absorption, particle size reduction (20–30 nm)
Menazea et al. [129]	Ag NPs	Laser wavelength (800 nm) Pulse width: 40 fs Power 1 mJ PRR: 1 KHz Ablation time: 15 min	Antibacterial efficiency, structural & optical properties	Spherical-shaped NPs, uniform size distribution
Mostafa et al. [130]	CdO NPs	Pulse duration: 7 ns Energy per pulse: 80 mJ	Stability, morphology	Crystalline and spherical NPs of size 47 nm
Altowyan et al. [131]	Au-Ag NPs	Pulse duration: 7 ns Energy per pulse: 50, 150, 250 mJ	Effect of laser energy on nanostructure	(Au)Core-(Ag)shell nanostructure formation. Ag-Shell thickness increased with laser energy
Alluhaybi et al. [132]	Au NPs	Laser wavelength: 1064 nm Pulse width: 8 ns Fluence: 7.28, 17.03, 21.55 and 23.96 J/cm^2^	Structural, morphological, optical properties	Generation of spherical NPs (7–30 nm) An increase in ablation energy yielded a blue shift in absorbance, smaller particles (30.1 to 7.5 nm)
Ibarra et al. [133]	TiO_2_	Laser wavelength: 532 nm Pulse width: 10 ns Fluence: 0.65 J/cm^2^ Irradiation time: 45, 60, 75, 90 min	Optical properties, energy bandgap	The shift of diffraction peaks and bigger spherical nanoparticles with an increase in irradiation time, phase change of TiO_2_
Mendivil et al. [134]	Pd NPs	Laser wavelength: 1064 nm Pulse width: 10 ns Fluence: 40.5–8 J/cm^2^ Irradiation time: 45, 60, 75, 90 min	Morphology, nanostructure, the effect of fluence on size of nanoparticles	Spherical morphology, cubic crystalline nanostructure. Average diameter increased with reduction in fluence (17 ± 6 nm for 40.5 J/cm^2^. 24 ± 7 nm for 18 J/cm^2^ 27 ± 9 nm for 8 J/cm^2^)
Kupracz et al. [135]	Fe based NPs	Laser wavelength: 1064 nm Pulse width:6 ns PRR: 10 Hz Fluence: 9–21 J/cm^2^ Irradiation time: 2–32 min	Stability, composition	An increase in fluence incrementally changes the NP diameter. Longer irradiation and storage lead to agglomeration
Goncharova et al. [136]	Cu NPs	Laser wavelength: 1064 nm Pulse width: 7 ns PRR: 20 Hz	Structure, morphology, stability, composition	Cubic-shaped NPs formed initially, 10–50-nm size range, nanoribbons formed after 2 weeks
Altuwirqi et al. [56]	Al NPs	Laser wavelength: 532 nm Ablation time: 15 min PRR: 10 Hz Pulse width: 6 ns	Structure, morphology	Spherical morphology, core-shell nanostructure formation Average diameter: 12 ± 9 nm
Riahi et al. [137]	Al NPs	Laser wavelength: 1064 nm Ablation time: 15 min PRR: 10 Hz Pulse width: 6–7 ns	Thermal conductivity, optical properties	Increased thermal conductivity of nanofluid. Change in nanoparticle concentration
Nassar et al. [138]	Zn NPs	Laser wavelength: 800 nm Ablation time: 10 min PRR: 1 KHz Pulse width: 130 fs	Effect of pulse energy (0.05 mJ, 1.11 mJ, 1.15 mJ) on NP size and distribution	NP size increases with pulse energy Higher absorption

**Table 3:** State of the art studies conducted to examine the influence of liquid media on nanoparticle formation.

Reference	Liquid Medium	Study	Formed Nanostructure, Morphology	Research Outcome
Lee et al. [153]	Methanol, DIW, hexane, acetonitrile	Cavitation bubble dynamics of Ni NPs	FCC/HCP, Pure FCC and spherical-shaped NPs	Bubble lifetime and crystal structure depends on liquid media
Solati et al. [154]	Distilled water, acetone, CTAB	Effect of liquid environment on the properties of TiO_2_	Polycrystalline, spherical-shaped NPs	Distilled water produces smaller, narrow size distribution, better adhesion than other solvents
Moura et al. [155]	DDW, acetone and ethanol	Study characteristics of Ag NPs	Spherical NPs	Liquid media play a major role in the mean size and size distribution. Acetone and ethanol resulted in low productivity but a bigger NP size.
Lasemi et al. [156]	Distilled water, ethanol, butanol, iso-propanol	Study the development of Ni, Fe and W NPs	Not reported	Ni showed more incubation than other metals.
Ali et al. [157]	DIW, propanol	Study the characteristics, mechanical and structural surface changes in Ti NPs	Nanocones, -globules in DIW Dendritic, globular in propanol	Ablation mass and nanostructure formation and bubble confinement are dictated by the liquid medium
Lee et al. [158]	DIW, methanol, hexane, acetonitrile	Study chemical reactivity of Au, Au-GC NPs in various solvents	Spherical, agglomerated chains and polycrystalline nanostructure	The enhanced catalytic activity of Au NPs
Nikov et al. [159]	Chloroform, toluene and ethanol	Study on the effect of optical properties and size distribution in different solvents for Au NPs	Spherical and spheroidal morphology, elongated nanostructures	Mean size distribution influenced by the liquid medium

## Data Availability

Not applicable.

## Abbreviations

A	absorbance
ε	molar absorbance coefficient
c	molar concentration
*l_0_*	optical path length
PLA	pulse laser ablation
NPs	nanoparticles
*D_T_*	thermal diffusivity
α	attenuation coefficient
*f*	focal length of the lens
*l*	thickness of the liquid layer
*n*	refractive index of liquid
*r*	radius of the laser beam before focus
*H*_v_	heat of vaporisation
ρ	density
CQDS	carbon quantum dots
PLAL	pulsed laser ablation in liquid
*λ*	wavelength
$\tau_{L}$	pulse duration
$l_{\alpha }$	optical penetration
*k*	thermal conductivity
$C_{P}$	specific heat capacity of the target material
*τ* *_e_*	electron cooling time
*τ* *_p_*	lattice heating time
$C_{e}$	volumetric heat capacity of electron
$T_{e}$	electron temperature
*C*	lattice volumetric heat capacity
$k_{e}$	electron thermal conductivity
γ	electron lattice energy heat transfer coefficient
*R*	reflectivity of the target material
*I*	laser intensity
*F*	laser fluence
*F_th_*	threshold fluence
*V*	scanning speed
*T*	pulse duration
*S*	laser beam spot size
*E*	pulse energy
PRR	pulse repetition rate
ps	picosecond
ns	nanosecond
fs	femtosecond
